# Quality of the Physical Education Teacher’s Instruction in the Perspective of Self-Determination

**DOI:** 10.3389/fpsyg.2021.708441

**Published:** 2021-07-20

**Authors:** Argenis P. Vergara-Torres, José Tristán, Jeanette M. López-Walle, Alejandra González-Gallegos, Athanasios Sakis Pappous, Inés Tomás

**Affiliations:** ^1^School of Sports Organization, Autonomous University of Nuevo León, San Nicolás de los Garza, Mexico; ^2^Department of Sport and Event Management, Business School, Bournemouth University, Bournemouth, United Kingdom; ^3^Faculty of Psychology, University of Valencia, Valencia, Spain

**Keywords:** task presentation, corrective feedback, legitimate perception, basic psychological needs, subjective vitality, physical education

## Abstract

The teacher’s instructions in physical education class have important implications for the psychological well-being of their students. The aim of this study was to analyze, under the postulates of the Self-Determination Theory (SDT), a model with the following sequence: the perception of the quality of the instructions (task presentation, amount of corrective feedback, and its legitimate perception) generated by the physical education teacher, the satisfaction of the three basic psychological needs and the subjective vitality in young students. The participants were 890 students (462 males and 428 females) of primary level from the metropolitan area of Monterrey, Mexico, between ages 11 and 13 (*M* = 11.36; *SD* = 0.49). The structural equation modeling showed positive and significant associations in all model interrelations, that is, task presentation and the amount of corrective feedback (*B* = 0.88, *p* < 0.001), and this in turn with legitimate perception (*B* = 0.81, *p* < 0.001); the legitimate perception of feedback and the satisfaction of the need for autonomy, competence, and relatedness (*B* = 0.63, *p* < 0.001; *B* = 0.90, *p* < 0.001; *B* = 1.01, *p* < 0.001, respectively); finally, the satisfaction of the three psychological needs and the subjective vitality (*B* = 0.12, *p* < 0.01; *B* = 0.43, *p* < 0.001; *B* = 0.24, *p* < 0.001, respectively). Therefore, the importance of a quality task presentation, as well as providing corrective feedback based on support for autonomy, is evident, so that students perceive it legitimately and thus facilitate the satisfaction of their basic psychological needs and in consequence, indicators of psychological well-being such as subjective vitality.

## Introduction

In the instructional process, it is expected that significant changes are generated in the behavior of learners. In this interaction, students experience sensations and perceptions that can affect their behavior and emotions either favorably or unfavorably, depending on the social context that the teacher promotes by providing a sense of structure (Mageau and Vallerand, 2003; Haerens et al., 2013; Curran and Standage, 2017; Ryan and Deci, 2017) or task presentation and corrective feedback (Tristán et al., 2016). In the context of physical education, teacher’s instructions refer to quality task presentation (Tristán et al., 2016) or structure before the activity (Haerens et al., 2013). Firstly, this involves communicating with students the meaning and importance of what is to be learned as well as organizing students, spaces, equipment, and times to practice (Tristán et al., 2016). Secondly, it entails also providing corrective feedback or structure during the activity (Haerens et al., 2013) that is accepted or perceived as legitimate by learners (Vergara-Torres et al., 2020).

Quality task presentation and corrective feedback are among the most important behaviors in the instructional process in the context of physical education (Hall et al., 2011) and are variables that can be considered as predictors of pedagogical effectiveness (Hall et al., 2011; Rink, 2019). Therefore, it is important for the teacher to develop the ability to present tasks in a way that allows students to feel able to begin to engage in learning tasks (Garza-Adame et al., 2017; Tristán et al., 2019) and to provide corrective feedback that allows students to maintain a sense of confidence in improving their own mistakes. In this way, feelings of incompetence in the tasks they perform will be avoided, even though the corrective feedback contains information of poor performance (Mouratidis et al., 2010; Vergara-Torres et al., 2020).

The topic of teachers’ instructions in the physical education classroom has been studied through the Self-Determination Theory (SDT; Ryan and Deci, 2017; for a review of studies based on SDT, see: Vasconcellos et al., 2019) and more specifically, through one of its mini-theories, the Basic Psychological Needs Theory (Deci and Ryan, 2000; Curran and Standage, 2017; Ryan and Deci, 2017). This theory postulates that human beings possess the universal psychological needs of *autonomy* (feeling that behavior and decisions originate from the inner self, that is, self-determination of one’s own behaviour), *competence* (experience of being able to perform effectively in context), and *relatedness* (bonding and attachment to others whose response is reciprocal). All three of them are essential for optimal physical, psychological, and social development of the organism (Ryan and Deci, 2017; Vansteenkiste et al., 2020).

Self-determination Theory also states that contextual factors (i.e., the physical education class) can influence personal factors (i.e., the satisfaction or frustration of basic psychological needs) and these, in turn, can influence the physical, psychological, and social well-being or discomfort (i.e., vitality, positive or negative effect, and life satisfaction) of students (Ryan and Deci, 2017). Through their instruction in the classroom, teachers can promote the satisfaction of basic psychological needs, and thus they can improve their students’ perception of well-being (Sierens et al., 2009; Curran and Standage, 2017; Tristán et al., 2019; Vergara-Torres et al., 2020).

From a pedagogical perspective, task presentation and corrective feedback can be conceptualized as phases of the instructional process (William and Hodges, 2005), while studies that have addressed instructional aspects from psychological perspectives have named these phases as structure before and structure during the activity (Haerens et al., 2013; Tristán et al., 2016), respectively. Structure has been studied as a single construct or as two dimensions: structure before and structure during activity (Haerens et al., 2013). Both cases relate to the social context and are associated with the autonomy-supportive and relationship-supportive style (Sierens et al., 2009; Curran et al., 2013; Haerens et al., 2013; Curran and Standage, 2017) of the teacher; as well as the satisfaction and frustration of basic psychological needs, engagement and disaffection (Curran et al., 2013; Curran and Standage, 2017), and self-regulated learning of the students (Sierens et al., 2009).

Previously, other studies have analyzed the relationship between task presentation (Tristán et al., 2019) and corrective feedback (Vergara-Torres et al., 2020) with the satisfaction of psychological needs and subjective vitality as an indicator of well-being in physical education students under the SDT. However, it seems to be unclear whether teachers who are able to deliver quality task presentations are also able to give corrective feedback that, far from hindering the satisfaction of psychological needs, favors it. In this regard, the work of Haerens et al. (2013) concluded that the two dimensions of structure were unrelated to each other, noting that structure before the activity had better scores. Similarly, the research by Chen et al. (2012) found that the teachers who participated in their study demonstrated that most of the time their task presentations were performed with quality, while the dimension of instructional response, which includes all the elements of structure during the activity, was only partially demonstrated.

These studies seem to indicate that, although the teachers in these studies are able to deliver quality presentations of the tasks, they do not provide adequate feedback during the activity. Furthermore, it is necessary to mention that there is evidence that, in order for corrective feedback not to have a negative impact on the satisfaction of basic psychological needs, it is necessary that it is given under certain characteristics that make students to see it as fair and justified, that is, that they have a legitimate perception of this feedback (Mouratidis et al., 2010; Vergara-Torres et al., 2020; Tristán et al., 2021). In this sense, the question arises as to whether teachers who are able to deliver quality task presentations are also able to provide corrective feedback that is perceived as legitimate by their students and, therefore, has a positive impact on their well-being, a question that this study aims to answer.

**Figure 1:**
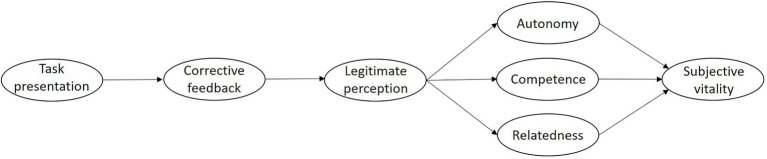
Hypothesized model.

Currently, the physical education classroom represents the only space in which children and adolescents can engage in physical activity throughout the week (Villagrán et al., 2010; Abarca-Sos et al., 2015). Therefore, it is particularly relevant to study the factors involved in optimal development and health in this context. In this study, and in accordance with previous studies that have found that quality in the presentation of tasks (Tristán et al., 2019) and corrective feedback are perceived as legitimate by students (Vergara-Torres et al., 2020), we consider that there may be a positive relationship between both variables, which, in turn, will have an impact on the satisfaction of psychological needs and greater perceptions of vitality in students. Taking all the above into account, the aim of this paper is to analyze a model that associates the relationships between task presentation and corrective feedback as variables associated with the teacher, and the legitimate perception of such feedback, the satisfaction of the three basic psychological needs and subjective vitality, as variables related to the students in the physical education class (see Figure 1).

## Materials and Methods

### Study Desing and Participants

This study is a cross-sectional, correlational study (Awang, 2012; Pandey and Pandey, 2015). Given the complexity of including all schools in the metropolitan area of Monterrey (Mexico), convenience sampling was used. The vast majority of the population in the area where the study was conducted are identified as Mexican-Latin American. The schools selected had in common that they were located in middle and lower-middle socio-economic areas. Participants were 890 students (462 males and 428 females) in sixth grade of primary school in the metropolitan area of Monterrey, Mexico, aged 11–13 years (*M* = 11.36; *SD* = 0.49), who had between one and two physical education classes per week with an average duration of 46.03 min (*SD* = 5.24).

Regarding the power analysis, it has been estimated that, assuming a small effect size (*f*^2^ = 0.02), for a maximum number of six predictors, and a type I error probability of 0.05, to achieve a statistical power of 0.80, a sample size of 688 individuals would be required (Faul et al., 2009). Therefore, this study, with 890 participants is considered as having sufficient statistical power to detect relevant relationships.

### Procedure

Approval was obtained from the Research Committee of the Faculty of Sport Organization. Prior to the start of data collection, permission for the application of the instruments was requested from the principals of the selected schools and a copy of the instruments was provided. In those schools where authorisation was obtained, informed parental consent was requested. Inclusion criteria were that the participant was a regular student at the school and had at least one physical education class per week taught by a physical education professional. The exclusion criteria were if the student had a cognitive disability that prevented him/her from answering the questionnaire consciously/autonomously. Data were collected between January and February 2020. According to the procedure established at least one researcher attended the group classrooms on a normal school-day. Students were informed of the aims of the study, the voluntary nature and anonymity of participation, as well as the non-existence of right and wrong answers, and asked to give honest answers. Only those students who presented the informed consent form signed by their parents participated in the study. Upon completion of the questionnaires, the researcher ensured that all items were answered, so there was no loss of sample due to incomplete questionnaires. The application of the instruments lasted approximately 25 min and the physical education teacher was not present. At all times during the study, the ethical protocols established by the *American Psychological Association* (APA) were followed.

### Measures

To measure task presentation, the Coach’s Task Presentation Scale (Tristán et al., 2016) adapted to the context of physical education (Tristán et al., 2019) was used. The instrument consists of 11 items with a Likert-type response option ranging from 1 (*Strongly disagree*) to 5 (*Strongly agree*), where an example item is: “*My teacher verbally explains to me the movement, task, exercise or activity to be performed*.”

In the assessment of corrective feedback and legitimate perception, we used a version adapted to the context of physical education (Vergara-Torres et al., 2020) of the Corrective Feedback Scale (Tristán et al., 2013), which comprised four subscales (amount of corrective feedback, legitimate perception, illegitimate perception, and opportunity to learn) with four items each (16 items in total) and Likert-type response option, ranging from 1 (*Strongly disagree*) to 5 (*Strongly agree*). For the purposes of this study, only the subscales of amount of corrective feedback and legitimate perception were used. An example item for the amount of corrective feedback subscale is: “*Is it true that your physical education teacher points out your mistakes?*,” while for the legitimate perception subscale an example item is: “*If my teacher points out my mistakes, I find that he/she has a fair reason for doing so*.”

In measuring the satisfaction of basic psychological needs, the Mexican Version of the Basic Psychological Needs Satisfaction in Physical Education Scale (Zamarripa et al., 2017) was used, which is composed of 16 items, divided into three subscales that assess the psychological needs of autonomy (six items), competence (five items), and relatedness (five items). The scale has Likert-type response options with ranges from 1 (*Strongly Disagree*) to 7 (*Strongly Agree*). As examples of items, for the autonomy subscale, it is: “*My opinion counts as to what activities I want to do*,” for the competence subscale: “*I think I am pretty good*,” and for the relatedness subscale: “*Supported*.”

Finally, to assess subjective vitality, an adapted version (Tristán et al., 2019; Vergara-Torres et al., 2020) of the Mexican Version of the Subjective Vitality Scale (López-Walle et al., 2012) was used. This scale is composed of six items with Likert-style response ranging from 1 (*Not true*) to 7 (*True*). An example of an item for this scale is: “*I feel encouragement and enthusiasm (alive) and full of life (vital)*.”

### Data Analysis

Descriptive analyses, reliability and correlation of each of the study variables were performed with SPSS version 25 software. The normality of the data was determined according to the criterion of skewness and kurtosis values in a range of −1, 1 (Muthén and Kaplan, 1985, 1992; Ferrando and Anguiano-Carrasco, 2010). The reliability of the instruments was determined through Cronbach’s alpha coefficient. The Pearson’s correlation test was used to determine the level of association between the variables studied.

To analyze the factorial structure of each of the study variables and the degree of association between them in the hypothesized model (see Figure 1), confirmatory factor analysis (CFA) and structural equation modeling (SEM) were used, respectively, using the Weighted Least Square Mean and Variance (WLSMV) estimation method, since a certain percentage of items were far from the normal distribution. Concretely, 42.8% of the items (15 out of 35 items) had skewness and/or kurtosis values lower than −1 or higher than +1 (Muthén and Kaplan, 1985, 1992; Ferrando and Anguiano-Carrasco, 2010).

To assess the fit of the models, the following goodness-of-fit indices were used: the Chi-square statistic (*χ*^2^), the Comparative Fit Index (CFI), the Tucker Lewis Index (TLI), and the Root-Mean-Square-Error of Approximation (RMSEA; Hu and Bentler, 1999; Kline, 2005). The CFA, structural equation modeling, and indirect effects analysis were performed using Mplus 8 (Muthén and Muthén, 1998–2010).

The hypothesized mediation model included all the study hypotheses. To test the mediated or indirect effects involved in the mediation model, it was used the bias-corrected (BC) bootstrap confidence interval method (MacKinnon et al., 2004; William and MacKinnon, 2008) as implemented in Mplus (Lau and Cheung, 2012). This method involves the calculation of the product of the regression coefficients that estimate the indirect effects (*abc*_1_*d*_1_; *abc*_2_*d*_2_; and *abc*_3_*d*_3_), where *a* is the coefficient estimating the relationship between task presentation and corrective feedback, *b* is the coefficient estimating the relationship between corrective feedback and legitimate perception, *c*_1_, *c*_2_, and *c*_3_ are the coefficients estimating the relationship between legitimate perception and autonomy, competence, and relatedness, respectively, and *d*_1_, *d*_2_, and *d*_3_ are the coefficients estimating the relationship between autonomy, competence and relatedness (respectively), and subjective vitality. Then, the BC bootstrap confidence interval for the indirect effects (*abc*_1_*d*_1_; *abc*_2_*d*_2_; and *abc*_3_*d*_3_) is obtained. If the confidence interval does not include zero, the null hypothesis of no mediation is rejected, providing empirical support for the indirect effect.

## Results

### Descriptive Statistics

Table 1 presents the descriptive statistics for each of the study variables. According to the skewness and kurtosis coefficients, the data follow a normal distribution, being in the range of −1, 1. Regarding the reliability values, most of the scales were satisfactory, presenting values above 0.70 (Peterson, 1994). With the exception of the autonomy subscale, which, with a score of 0.67, is considered as adequate, so, together with the theoretical relevance of the variable, it was decided to keep it in the study. Finally, all the correlations were positive and significant (*r* values between 0.27 and 0.63; *p* < 0.01).

**Table 1:** Descriptive statistics, reliability, and correlation between study variables.

	*M*	*SD*	*α*	Skewness	Kurtosis	1	2	3	4	5	6
1.Task presentation	4.01	0.63	0.84	−0.79	0.59	-					
2.Corrective feedback	3.81	0.83	0.74	−0.67	0.26	0.56	-				
3.Legitimate perception	4.19	0.66	0.71	−0.78	0.44	0.50	0.63	-			
4.Autonomy	4.37	1.18	0.67	−0.08	−0.48	0.35	0.32	0.27	-		
5.Competence	5.13	1.20	0.79	−0.56	0.28	0.38	0.34	0.33	0.34	-	
6.Relatedness	5.38	1.36	0.90	−0.75	0.19	0.42	0.38	0.35	0.40	0.54	-
7.Subjective vitality	5.11	1.12	0.78	−0.58	−0.17	0.44	0.32	0.29	0.30	0.47	0.43

*M*, Mean; *SD*, Standard deviation; and *α*, Cronbach’s alpha; All the correlation are significant at level *p* < 0.01.

### Confirmatory Factor Analysis

Table 2 shows the goodness-of-fit indices for each of the scales and subscales used in the study. Since the obtaining values above the recommended 0.90 for the CFI and TLI (Hu and Bentler, 1999; Kline, 2005) and below 0.08 for the RMSEA (Browne and Cudeck, 1992), it was concluded that all the subscales presented a good fit.

**Table 2:** Goodness-of-fit indices for study variables.

Subscale	*χ* ^2^	*df*	CFI	TLI	RMSEA
Task presentation	133.74	40	0.96	0.95	0.05
Corrective feedback	2.45	2	0.99	0.99	0.01
Legitimate perception	6.79	2	0.99	0.97	0.05
Autonomy	35.74	7	0.97	0.95	0.06
Competence	14.86	4	0.99	0.98	0.05
Relatedness	6.74	3	0.99	0.99	0.03
Subjective vitality	17.86	7	0.99	0.98	0.04

### Structural Equation Modeling

**Figure 2:**
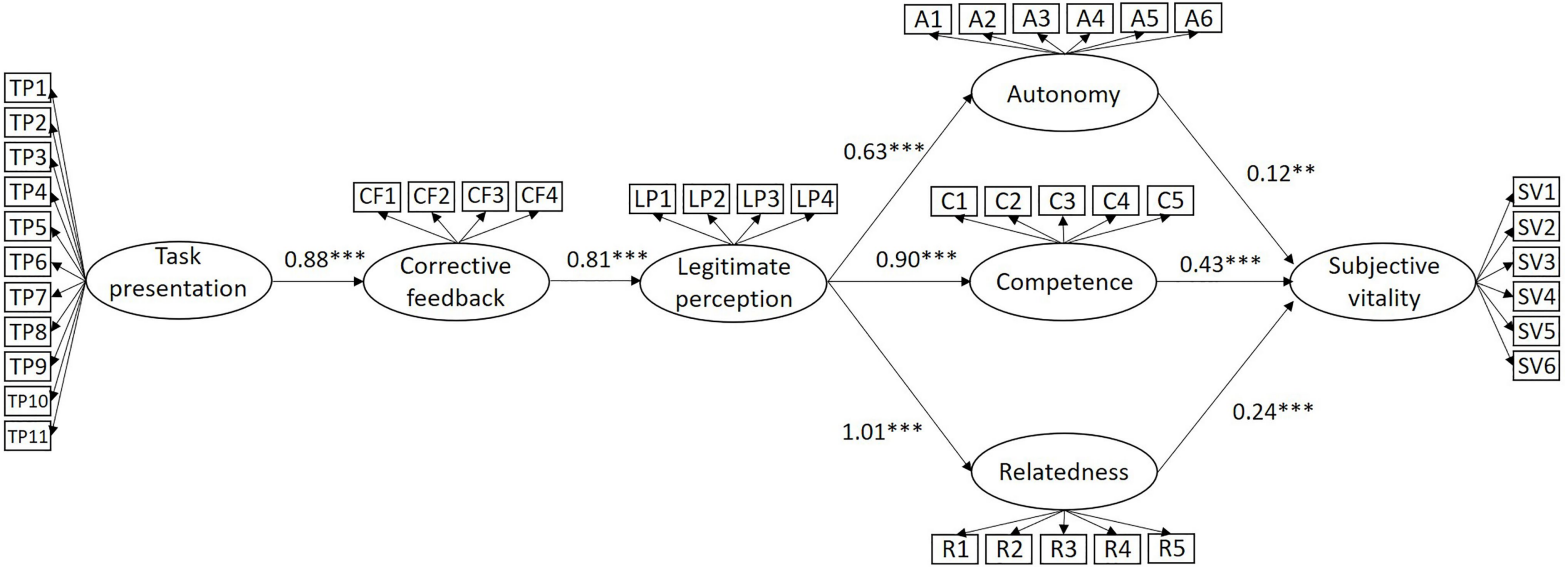
Non-standardized solution of the hypothesized model. ^∗∗∗^*p* < 0.001; ^∗∗^*p* < 0.01.

To determine the coefficient of association between the variables in the hypothesized model, a structural equation modeling analysis was performed. The model presented a satisfactory fit (*χ*^2^ = 3137.20, *df* = 771, *p* < 0.001; CFI = 0.92; TLI = 0.91; RMSEA = 0.06). Figure 2 presents the non-standardized solution of the model, in which a positive and significant association (*B* = 0.88, *p* < 0.001) can be observed between task presentation and corrective feedback, and a positive and statistically significant association was also found between corrective feedback and legitimate perception (*B* = 0.81, *p* < 0.001). Legitimate perception was positively and significantly associated with the basic psychological needs of autonomy (*B* = 0.63, *p* < 0.001), competence (*B* = 0.90, *p* < 0.001), and relatedness (*B* = 1.01, *p* < 0.001) and, finally, all three psychological needs were positively and significantly associated with subjective vitality (*B*_autonomy_ = 0.12, *p* < 0.01; *B*_competence_ = 0.43, *p* < 0.001; *B*_relatedness_ = 0.24, *p* < 0.001).

### Basic Psychological Needs Mediation

The BC bootstrap CI for the estimated mediated effects [*abc*_1_*d*_1_ = 0.05; CI 95% = (0.01, 0.11); *abc*_2_*d*_2_ = 0.28; CI 95% = (0.21, 0.35); *abc*_3_*d*_3_ = 0.17; CI 95% = (0.12, 0.23)] did not include zero. Therefore, it was concluded that corrective feedback, legitimate perception, and the basic psychological need (autonomy, competence, and relatedness) mediated the relationship between task presentation and subjective vitality.

With the purpose of further testing the aforementioned mediation effects, a partial mediation model (M2) was tested in which a direct path from task presentation to subjective vitality was added. The hypothesized model (M1) was compared with the partial mediation model (M2) that included the direct path from task presentation to subjective vitality. To compare the mediation models, differences between the chi-square statistics for the two nested models (M1 and M2) were estimated. The difference between the chi-square statistics provided by the two alternative models was statistically significant (Δ*χ*^2^ = 66.54, Δ*df* = 1, *p* < 0.01). Furthermore, the relationship between task presentation and subjective vitality was statistically significant (*B* = 0.26, *p* < 0.01). Thus, it was chosen M2 as the best fitting model and concluded that partial mediation was supported.

## Discussion and Conclusion

The main purpose of the present study was to analyze, under the postulates of SDT (Ryan and Deci, 2017), a model that associated the existing relationships between task presentation and corrective feedback as variables linked to the teacher’s quality instruction, and the legitimate perception of such feedback, the satisfaction of the three basic psychological needs and subjective vitality, as variables related to the well-being of students in the physical education class.

The results obtained showed a positive and significant association between task presentation and perceived corrective feedback, which is different from that reported by Chen et al. (2012) and Haerens et al. (2013). This provides empirical evidence that, when teachers are able to perform task presentations with quality, they are also able to provide an adequate amount of corrective feedback to their students during task practice. Previously, the work of Tristán et al. (2019) and Vergara-Torres et al. (2020) found that the teachers participating in those studies demonstrated quality task presentations and provided an adequate amount of corrective feedback, respectively. This allowed us to hypothesize that there could be a positive association between both behaviors, which, according to these results, could be corroborated.

In the same way, a positive and significant association was found between the corrective feedback provided by the teacher, and the legitimate perception of such feedback by the students, which is in accordance with what was hypothesized and reported in studies in the field of physical education (Vergara-Torres et al., 2020) and sport (Mouratidis et al., 2010; Tristán et al., 2021). This indicates that the student participants in this study had a high degree of acceptance of corrective feedback, and furthermore, it seems to reaffirm the previous finding by demonstrating that when teachers demonstrate quality task presentations, they are also able to provide corrective feedback under characteristics that allow their students to perceive it as fair and justified, that is, as legitimate.

On the other hand, legitimate perception, as hypothesized and reported in previous studies in the context of physical education (Vergara-Torres et al., 2020) and sport (Ríos, 2015; Tristán et al., 2021) was positively and significantly associated with the basic psychological needs of autonomy, competence, and relatedness. This result suggests that when students perceive the corrective feedback they have received as fair, reasonable, and justified, then they experience a greater sense of choice regarding the activities to be performed and the way of working to correct their mistakes. They also experiment a greater sense of ability and efficiency to perform the tasks and exercises, as well as a feeling of being related and belonging to a group. The literature has made clear the relevance of the satisfaction of basic psychological needs in the physical and psychological health of physical education students (Vasconcellos et al., 2019; Piñeiro-Cossio et al., 2021), so it is essential for the teacher to ensure that their students have legitimate perceptions of the corrective feedback granted.

To achieve this, the teacher should consider several guidelines. First, to satisfy the need for autonomy, when presenting learning tasks, the teacher should explain the relevance of the activities (Reeve and Jang, 2006; Curran et al., 2013; Curran and Standage, 2017) and their relation to prior knowledge (Chen et al., 2012). As well as showing sincere interest in their students’ preferences, listening to their opinion (Haerens et al., 2013; Reeve and Cheon, 2016) and providing opportunities for them to take initiative and make their own decisions (Reeve and Jang, 2006; Moreno-Murcia et al., 2012). Similarly, when providing corrective feedback, the teacher should do so in an autonomy-supportive style (Reeve, 2006; Mouratidis et al., 2010; Carpentier and Mageau, 2013, 2016) and thus be perceived legitimately by their students (Mouratidis et al., 2010). As well as offering a variety of choices to correct the problem (Deci et al., 1994; Mageau and Vallerand, 2003; Carpentier and Mageau, 2013, 2016) or providing fair and reasonable explanations in case it is a limited option (Henderlong and Lepper, 2002; Mouratidis et al., 2010).

Furthermore, to satisfy the need for competence, the teacher should in his or her task presentation, to provide clear expectations and task objectives (Haerens et al., 2013; Tristán et al., 2019), and to explain and demonstrate how to perform the activity effectively by focusing on the critical elements of the task (Chen et al., 2012; Smith et al., 2015; Tristán et al., 2016). When providing corrective feedback, the teacher should do so in an autonomy-supportive style (Reeve, 2009; Mouratidis et al., 2010; Carpentier and Mageau, 2013, 2016) in order to have high levels of perceived legitimacy (Mouratidis et al., 2010). It is also important that when providing corrective feedback, teachers should use familiar and informative language (Hattie and Timperley, 2007; Mouratidis et al., 2010; Reeve and Cheon, 2016), focusing on the error (Henderlong and Lepper, 2002) and attributing it to the strategy employed rather than lack of skill (Deci et al., 1994; Henderlong and Lepper, 2002), and providing sufficient time to work on the error (Henderlong and Lepper, 2002; Hattie and Timperley, 2007).

Finally, to satisfy the need for relatedness, during task presentation, it is necessary for the teacher to provide warm, friendly, and caring guidance and attention to all the students (Haerens et al., 2013; Smith et al., 2015; Curran and Standage, 2017; Ryan and Deci, 2017) and to involve them in explanations and demonstrations (Cox and Williams, 2008; Soenens et al., 2009; Rink, 2019). Similarly, when providing corrective feedback, the teacher needs to do so in an autonomy-supportive style (Reeve, 2009; Mouratidis et al., 2010; Carpentier and Mageau, 2013, 2016) that allow students to perceive it as legitimate (Mouratidis et al., 2010); transmitting it in an empathetic way, with an appropriate tone of voice (Carpentier and Mageau, 2013, 2016), enabling the student to rejoin the group if the tasks are group tasks and that the student, despite having received corrective information, does not stop feeling part of the group (Haerens et al., 2013).

In the case of the relationship between basic psychological needs and subjective vitality, a positive and statistically significant association was found. This is in accordance with what has been hypothesized and with what has been proposed by the SDT (Ryan and Deci, 2017), as well as with previous studies in the area of physical education (Tristán et al., 2019; Vergara-Torres et al., 2020) and sport (Garza-Adame et al., 2017). The evidence obtained from this research indicates that, when students in physical education class perceive themselves to be more autonomous, competent, and related to their peers and teacher, they feel more vital.

On the other hand, this research had some limitations. First, due to the complexity of coverage of all the schools that could be selected in a representative sample in the metropolitan area of Monterrey (Mexico), convenience sampling was used, in addition to only having students in the sixth grade of primary school, which means that these results cannot be extrapolated to other populations. Future studies could include older and younger students to determine whether the variables studied behave in the same way. Second, the cross-sectional nature of this study is a limitation when it comes to establishing cause-effect relationships between the variables analyzed. Future studies would allow further analysis of the variables in this study with longitudinal designs. Third, all data were collected using self-report questionnaires. It is suggested, therefore for future studies, to use other types of data collection techniques regarding teacher intervention (e.g., teacher-answered questionnaires or rubrics completed by an external evaluator) and to extend the study variables associated with quality in physical education teaching (e.g., task design, classroom organization, among others).

In conclusion, when physical education teachers provide quality task presentations, they are also able to give corrective feedback that is perceived as legitimate by their students and therefore this helps students to maintain a sense of confidence in improving their own mistakes and not to feel incompetent in the learning tasks, they perform even though the corrective feedback contains information of poor performance. In addition, task presentation and an adequate amount of legitimately perceived corrective feedback favors the satisfaction of basic psychological needs, and these in turn promote higher perceptions of subjective vitality in physical education students.

## Practical Applications

The findings resulting from this study can be used in the training of pre-service and in-service physical education teachers and thus contribute to achieving quality instructional practices that facilitate the creation of environments that allow their students to experience psychological well-being. Similarly, the findings and guidelines raised throughout the manuscript can be used for the development and validation of scales or rubrics to assess teacher instruction before and during class, and thus, explore aspects that can contribute to understanding the possible complications and strengths of teachers to achieve teaching practices of excellent quality.

## Data Availability Statement

The raw data supporting the conclusions of this article will be made available by the authors, without undue reservation.

## Ethics Statement

The studies involving human participants were reviewed and approved by the Comité de investigación de la Facultad de Organización Deportiva. Written informed consent to participate in this study was provided by the participants’ legal guardian/next of kin.

## Author Contributions

AV-T, JT, JL-W, and IT contributed to conception and design of the study. AV-T, AG-G, and IT organized the database and performed the statistical analysis. AV-T and JT wrote the first draft of the manuscript. AV-T, JT, IT, JL-W, and AP wrote sections of the manuscript. AP and AG-G contributed to global review of the article and its methodology and the accuracy of the translation. All authors contributed to manuscript revision, read and approved the submitted version.

### Conflict of Interest

The authors declare that the research was conducted in the absence of any commercial or financial relationships that could be construed as a potential conflict of interest.
