# Corrigendum: Quality of the Physical Education Teacher's Instruction in the Perspective of Self-Determination

**DOI:** 10.3389/fpsyg.2021.749678

**Published:** 2021-09-20

**Authors:** Argenis P. Vergara-Torres, José Tristán, Jeanette M. López-Walle, Alejandra González-Gallegos, Athanasios Sakis Pappous, Inés Tomás

**Affiliations:** ^1^School of Sports Organization, Autonomous University of Nuevo León, San Nicolás de los Garza, Mexico; ^2^Department of Sport and Event Management, Business School, Bournemouth University, Bournemouth, United Kingdom; ^3^Faculty of Psychology, University of Valencia, Valencia, Spain

**Keywords:** task presentation, corrective feedback, legitimate perception, basic psychological needs, subjective vitality, physical education

In the original article, there was a mistake in Figure 2 as published. The text in the figure appears in Spanish and not in English. The corrected Figure 2 appears below.

**Figure 2:**
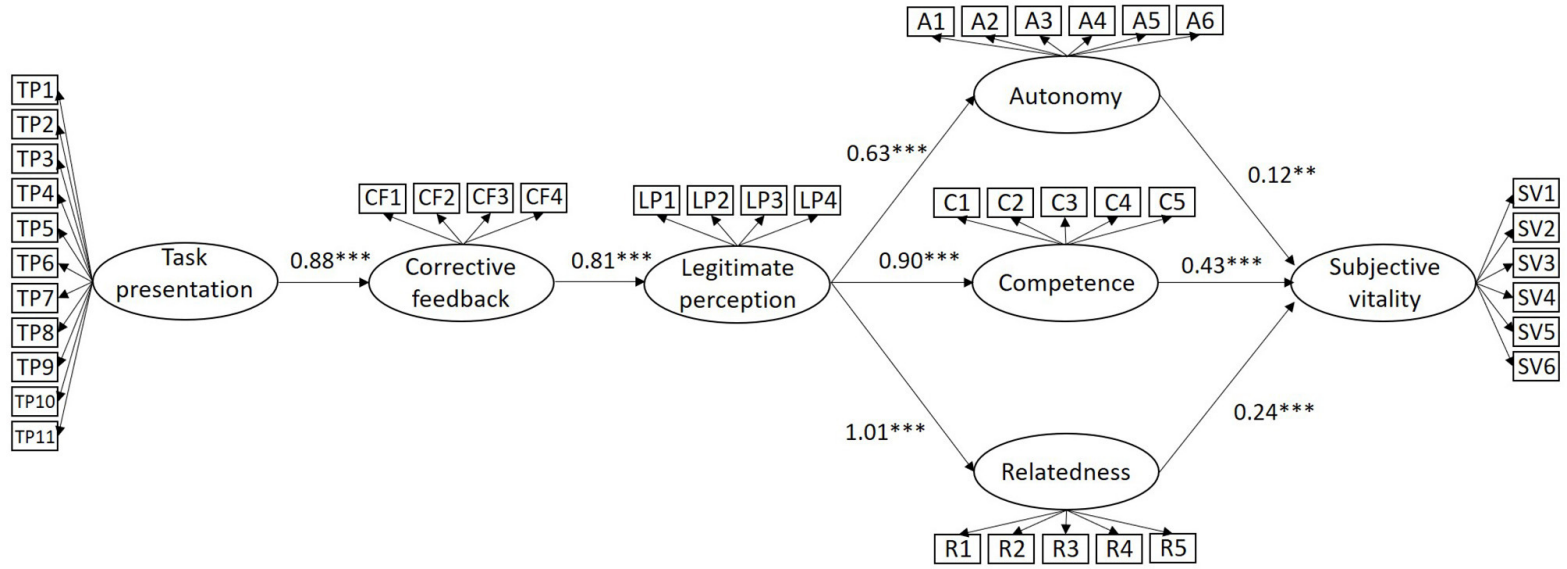
Non-standardized solution of the hypothesized model. ***p < 0.001; **p < 0.01.

The authors apologize for this error and state that this does not change the scientific conclusions of the article in any way. The original article has been updated.

## Publisher's Note

All claims expressed in this article are solely those of the authors and do not necessarily represent those of their affiliated organizations, or those of the publisher, the editors and the reviewers. Any product that may be evaluated in this article, or claim that may be made by its manufacturer, is not guaranteed or endorsed by the publisher.

